# The Significance of the Cell-Mediated Host Immune Response in Syphilis

**DOI:** 10.3390/microorganisms12122580

**Published:** 2024-12-13

**Authors:** Konrad Kaminiów, Martyna Kiołbasa, Maciej Pastuszczak

**Affiliations:** Clinical Department of Dermatology, Medical University of Silesia, Marii Curie-Skłodowskiej 10, 41-800 Zabrze, Poland; martynakiolbasa@gmail.com (M.K.); maciej.pastuszczak@sum.edu.pl (M.P.)

**Keywords:** *Treponema pallidum*, syphilis, cell-mediated immune response, immunology, microbiology

## Abstract

Syphilis, caused by the highly invasive pathogen *Treponema pallidum*, remains one of the oldest and most significant public health challenges. According to the World Health Organization (WHO), the number of new syphilis cases among adults aged 15–49 years in 2022 was estimated at approximately 8 million, with notable increases observed in Europe, the Americas, and Africa. The cellular immune response plays a critical role in combating this infection, and its insufficient activity may contribute to chronic progression of the disease. *T. pallidum* effectively evades the host immune response, enabling its prolonged survival within the host and increasing the risk of late complications such as neurosyphilis and cardiovascular syphilis. This review article discusses the mechanisms of cellular immune responses in *T. pallidum* infection, including T lymphocyte activation, proinflammatory cytokine production, and the roles of macrophages and dendritic cells in pathogen recognition and elimination. Additionally, it examines the immune evasion strategies employed by *T. pallidum*, such as the low immunogenicity of its antigens and its ability to suppress the activation of effector cells. A comprehensive understanding of the current knowledge regarding cellular immune mechanisms may contribute to the development of more effective diagnostic and therapeutic approaches in syphilis management.

## 1. Introduction

Syphilis is a chronic, sexually transmitted infection caused by the bacterium *Treponema pallidum* [1,2]. Syphilis remains a significant epidemiological problem worldwide, particularly in developing countries, mainly in sub-Saharan Africa and Southeast Asia. According to the latest WHO estimates, the global incidence of syphilis is approximately 8 million new infections annually [3].

Interestingly, in recent decades, there has been a systematic increase in the number of cases in regions previously characterized by low prevalence, including Europe, North America, and Australia [2,4,5,6]. The highest risk of syphilis infection is observed among individuals engaging in risky unprotected sexual practices, those with multiple sexual partners, sex workers, men who have sex with men (MSM), and people living with HIV (PLWHIV) [2,7,8].

The primary pathogen causing syphilis is classified as a highly invasive bacterium. It enters the human body through exposure of skin microabrasions to skin lesions or bodily fluids containing *T. pallidum*. The bacterium multiplies locally while simultaneously spreading through the bloodstream and lymphatic vessels [9,10,11]. Such infections most commonly occur through sexual contact but can also occur via transplacental transmission (e.g., from an infected mother to her child). Syphilis not only affects the genitourinary system but also impairs other systems, including the neurological, cardiovascular, ocular, and hepatic systems [12,13].

Considering that *T. pallidum* does not produce cytotoxins, and no other virulence factors for this bacterium have been identified so far, it seems likely that the organ and tissue damage observed in syphilis is solely the result of an acute nonspecific inflammatory response followed by an induced specific immune response.

For years, the cellular immune response has been suggested to play a significant role in the pathophysiology of syphilis. Current knowledge indicates that the cellular response that develops in the early stages of syphilis infection eliminates bacteria from their entry site, clinically manifesting as spontaneous resolution of symptoms. Unfortunately, the mechanisms through which some individuals achieve self-healing after symptom resolution while others experience transition to a latent state remain unknown.

*T. pallidum* can evade the immune response, allowing it to persist and spread within the host. This is due to the spirochete’s adaptive abilities, including antigenic variability and a capacity to hide from the host immune system. These features make the study of cellular responses particularly important [14].

In response to *T. pallidum* infection, T cells play a key role in initiating and coordinating immune reactions. Helper T cells (Th1 and Th2) and cytotoxic T lymphocytes (CTLs) are particularly significant, as they participate in eliminating infected cells. The Th1 response, characterized by the secretion of cytokines such as interferon-gamma (IFN-γ), is crucial for limiting pathogen spread. Studies show that individuals with a well-developed Th1 response are more likely to control infection and limit disease progression [15].

Conversely, an ineffective immune response can lead to chronic infection and the development of advanced stages of the disease, such as tertiary syphilis [16]. Therefore, understanding the interactions between *T. pallidum* and the host immune system—particularly the cellular response—is not only of theoretical importance but could also lead to the development of more effective therapeutic strategies.

## 2. Characteristics of the Pathogen and Mechanisms of Immune Evasion by *Treponema pallidum*

*Treponema pallidum* subspecies pallidum is a Gram-negative, spiral-shaped bacterium measuring approximately 0.2–0.4 μm in diameter and 6–20 μm in length. It belongs to the order *Spirochaetales*, family *Spirochaetaceae*, genus *Treponema*, and species *Treponema pallidum*. Within the *Treponema pallidum* species, three subspecies are distinguished: (1) *T. pallidum subspecies pallidum*—the causative agent of syphilis, (2) *T. pallidum subspecies pertenue*—the etiological agent of yaws, and (3) *T. pallidum subspecies endemicum*—the etiological agent of endemic syphilis.

These species (*T. pallidum* subsp. *pallidum*, as well as *T. pallidum* subsp. *pertenue*), despite being morphologically identical, as they cannot be distinguished by fluorescence or treponemal tests [17], and similarly unsuitable for cultivation under laboratory conditions and differ in pathogenicity, clinical manifestations, and the immune response they provoke. Researchers have examined genes in an effort to demonstrate differences between *T. pallidum* subsp. *pallidum* and other subspecies of *T. pallidum*. In each spirochete, the gene encoding the lipoprotein TpN15 is identical [18]. However, detailed molecular studies showed that *T. pallidum* subsp. *pallidum* differs from *T. pallidum* subsp. *pertenue* and *endemicum*, mainly due to a sequence difference in the region upstream of the gene that introduces a cleavage site for the restriction enzyme Eco47III [19] and a sequence change in the *gpd* gene that confers another cleavage site for the restriction enzyme Eco47III [20]. These unique restriction sites provide the genetic ability to distinguish syphilis spirochetes from the rest, as pathogenic subspecies of *T. pallidum* are morphologically identical by microscopy, and syphilis, yaws, and bejel infections induce multiple cross-reactive antibodies, making serological differentiation impossible. There are obvious issues that differentiate these diseases regarding the route of transmission—transmission of yaws and syphilis endemicum occurs through physical contact mostly at a young age, while syphilis has no age restriction and is generally transmitted through sexual contact (with the exception of congenital syphilis) [17]. The early stages of yaws and syphilis have some similarities, but late lesions of yaws are thought to be limited to the skin, bones, and joints, while syphilis, after the early cutaneous stage, can involve various tissues and organs [17].

It should be mentioned that yaws lesions tend to appear later and persist longer than syphilitic lesions. Interestingly, it has also been shown that, unlike *T. pallidum* subsp. *pallidum*, yaws is not characterized by placental transmission [21,22].

Another differentiating factor between *T. pallidum* subspecies may be the kinetics of the immune response. In preclinical studies on yaws, antibody titers peaked between 3 weeks (IgM) and 6 weeks (IgG). Titers dropped sharply at 3 months post-infection, while in animals infected with comparable *T. pallidum* subsp. *pallidum* inoculum, the highest antibody titers were observed at about 3 months and remained at similarly high levels for 5 months post-infection [23].

*T. pallidum* is an anaerobic bacterium with a very small genome (approximately 1.14 million base pairs), which limits its metabolic capabilities, resulting in a prolonged division time of up to 33 h. This makes it highly dependent on its host. The bacterium requires adequate moisture to survive and dies within a few hours outside the host organism. Humans are the only known natural hosts of *T. pallidum*. The bacterium is also highly sensitive to high temperatures, antiseptics, and detergents. These factors have so far precluded the development of an in vitro culture method for *T. pallidum*, significantly hindering laboratory research on this pathogen.

Numerous *T. pallidum* proteins activate macrophages and dendritic cells (DCs) through CD14- [24,25,26] and Toll-like receptor (TLR1 and TLR2)-dependent signaling pathways [24,26]. These pathogen-associated molecular patterns (PAMPs) are thought to be the main pro-inflammatory agonists during treponema infection [24]. However, *T. pallidum*, unlike other Gram-negative bacteria that have lipopolysaccharides, has a unique outer membrane (OM) structure that does not contain lipoproteins exposed on the surface. As a result, these PAMPs are not readily accessible to TLRs or other pattern recognition receptors (PRRs) on monocytes/macrophages or DCs [24,27,28,29]. The absence of LPS reduces detection of the bacterium by the immune system, allowing it to infiltrate and spread in the host without activating innate pathogen recognition systems.

Additionally, the outer membrane of *T. pallidum* contains several proteins critical to its pathogenicity. The bacterium’s outer membrane proteins (OMPs) have an exceptionally low density [9,24,27,30,31], and the antibodies they elicit in humans are limited [30,31]. As such, anti-treponemal antibodies alone may not suffice to control bacterial replication or prevent its dissemination [32]. Ex vivo opsonophagocytosis assays using rabbit peritoneal macrophages [33] or human PBMCs [34] have shown that a significant number of treponemes evade phagocytosis even in the presence of syphilitic immune sera.

Moreover, these OMPs are involved in various functions, including nutrient acquisition and adhesion to host cells. One of the best-studied OMPs is TprK, which exhibits antigenic variation in seven variable (V) regions of the *tprK* gene. This allows the bacterium to alter its surface proteins and evade the host immune response in syphilis patients [7,35]. This antigenic variation is a key factor in the bacterium’s ability to cause chronic infections, as it can frequently and indefinitely alter its surface antigens to avoid detection [7,35]. Interestingly, some studies suggest that, during latency, treponemes reside in hair follicles and nerves [7,36], which may explain the reactivation of chronic latent infections, leading to tertiary syphilis in the pre-antibiotic era [7,37].

A study by Xia W. et al. [38] of 28 individuals demonstrated another mechanism by which *T. pallidum* evades the host immune response [38]. The researchers measured the activity of caspase-1 and caspase-3, well-known mediators of programmed cell death, and found that *T. pallidum* infection induced programmed death of CD4+ and CD8+ T cells via both pyroptosis and apoptosis, thereby impairing a robust immune response [38].

In a study conducted by Babollin et al. [39], an oligomeric protein from the bacterioferritin family produced by *T. pallidum* (TpF1) was analyzed [39]. The authors observed elevated serum antibody levels against TpF1 in patients with secondary syphilis, correlating with a significantly higher average percentage of T regulatory cells (Tregs) in infected patients compared to healthy controls [39]. Tregs are a unique subpopulation of T cells that suppress immune responses, thereby contributing to the persistence of chronic syphilitic disease [39]. Additionally, TpF1 promotes vasculitis, angiogenesis, and cardiovascular complications in patients with secondary and tertiary syphilis [40].

*T. pallidum* likely also utilizes enzymes such as hyaluronidase to degrade the extracellular matrix (ECM), facilitating its invasiveness and ability to penetrate tissues [32].

These processes collectively enable *T. pallidum* to efficiently infect the host, evade the immune system, and spread throughout tissues.

## 3. The Course of Infection and the Mechanisms of the Cell-Mediated Host Immune Response Accompanying It

*T. pallidum* infection represents a continuous balance between evasion of the immune response and recognition of the bacteria by host immune cells. Evidence suggests that spirochetes circulate in the blood largely unchallenged by the host’s immune defenses. It is only the significant spirochetal burden present in the skin that triggers a highly complex inflammatory cellular immune response, which paradoxically does not effectively control the rapid replication of the spirochetes [24]. The cytokines and the phenotypes of cells present in early syphilitic lesions in both human infections and experimental infections in rabbit models suggest that the early immune response to *T. pallidum* is characteristic of delayed hypersensitivity or a Th1 response, involving a strong cellular component [8,41]. However, the extent to which the various cellular components of syphilitic infiltrates contribute to clearing spirochetes remains an open question.

After penetrating the skin or a mucous membrane, *T. pallidum* rapidly enters deeper tissues and the bloodstream [9]. One element of the nonspecific response associated with local *T. pallidum* infection is the stimulation of skin cells to produce matrix metalloproteinase 1, which breaks down collagen and facilitates bacterial penetration into deeper tissue layers [42]. Additionally, *T. pallidum* induces the expression of adhesion molecules (ICAM-1, VCAM-1) on endothelial cells, enabling inflammatory cells to migrate from the bloodstream to infected tissues [43].

The predominance of sub-surface treponemal proteins [10,44], along with the previously mentioned lack of pro-inflammatory lipopolysaccharides (LPS) and lipooligosaccharides (LOS) [45], allows the bacteria to spread silently, accompanied by low inflammation and systemic symptomatology, as the innate immune system barely detects them [7]. As observed in the primary lesion (hard chancre), the initial step in the host response to *T. pallidum* involves the formation of opsonizing antibodies that facilitate the internalization and degradation of treponemes through phagocytosis [7]. Phagosomes containing ingested treponemes subsequently fuse with lysosomes to form phagolysosomes, where the treponemes are degraded. Lipopeptides released in phagolysosomes trigger the recruitment of T cells [7]. In a rabbit model of syphilis, T cells were observed at the site of bacterial entry as early as day 3 post-infection. The peak numbers of these cells in tissues occurred between days 10 and 13 post-infection and closely correlated with the number of replicating spirochetes at the site of inoculation [46,47].

Each ulcer containing replicating treponemes generates a complex inflammatory response comprising macrophages, T cells, and plasma cells. Locally activated CD4+ and CD8+ T cells (with CD4+ T cells predominating in early infection) mediate bacterial clearance primarily through the production of IFN-γ, which stimulates macrophages to internalize and degrade treponemes and triggers an inflammatory cascade [41]. Subsequently, macrophages engulf and kill opsonized spirochetes [41]. Between days 6 and 10 post-infection, a significant increase in macrophages in the cellular infiltrate is noted, with the peak number observed between days 13 and 17. During this period, a marked reduction in the number of treponemes in the affected tissue is observed [46,47]. Therefore, the primary response to *T. pallidum* infection is a predominantly Th1 cell-based immune response.

A study analyzing cytokine mRNA in human primary and secondary syphilitic lesions found that cytokines associated with Th1 (IFN-γ, IL-2, and IL-12) were present, while Th2-associated cytokines (particularly IL-4) were consistently absent [41,48]. A study by Arroll et al. [49] provided further evidence of Th1 predominance during early infection. The authors demonstrated that in vitro restimulation with whole *T. pallidum* antigens or recombinant treponemal antigens primarily induced the production of Th1-associated cytokine (IFN-γ and IL-2) mRNA in splenocytes from *T. pallidum*-infected rabbits [49]. Additionally, *T. pallidum* lipoproteins such as TpN17 and TpN47 are strong inducers of pro-inflammatory cytokines like IL-12, which can also promote the initiation of a Th1 response [41].

Due to the limited number of pathogen-associated molecular patterns (PAMPs) on its surface, serving as potent antigens to trigger an immune response, *T. pallidum* exhibits a high capacity for recurrence, which poses a challenge to the host’s innate immunity [41]. An example of this is the ease with which live treponemes spread during secondary and early latent syphilis, despite high levels of specific antibodies [41,50]. Secondary syphilitic lesions arise after treponemes traverse endothelial junctions, triggering a localized immune response consisting of macrophages, monocytes, and T cells [34,37,41,51].

**Figure 1:**
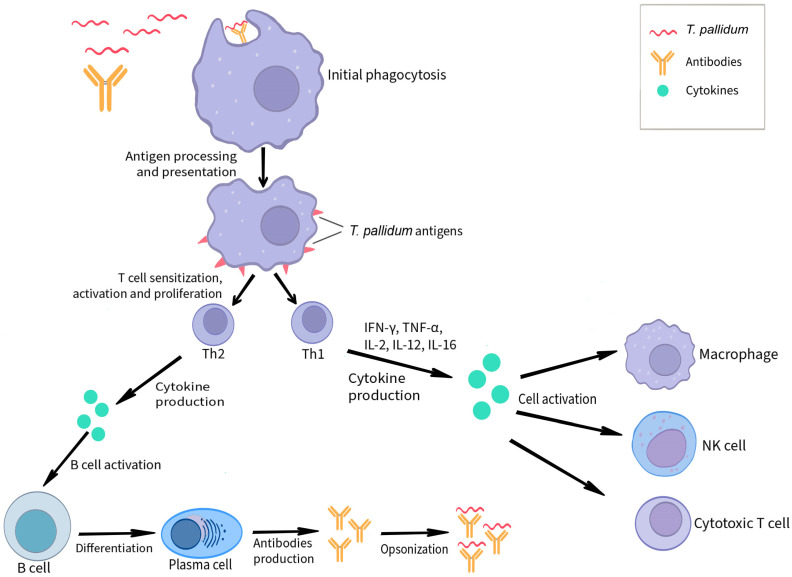
Scheme of the immune response to *T. pallidum* infection.

Figure 1 shows a simplified scheme of the immune response to *T. pallidum* infection.

### 3.1. Dendritic Cells

Dendritic cells, particularly their specialized subpopulation found predominantly in the skin and mucous membranes—Langerhans cells—appear to be the first critical link in the cellular immune response during *T. pallidum* infection. Treponemal lipoproteins, via toll-like receptors (TLR-2), stimulate dendritic cells. Pathogens are then phagocytosed by these cells, which subsequently migrate to lymph nodes to present bacterial antigens to specific T lymphocytes [52]. As mentioned earlier, treponemal lipoproteins that stimulate dendritic cells are not surface-exposed. This means that, for dendritic cells to initiate a nonspecific immune response, the bacteria must undergo at least partial degradation. Experimental syphilis models have shown that the time required to stimulate dendritic cells by *T. pallidum* is longer than that seen in other bacterial infections. The delay in stimulation, maturation, and migration of dendritic cells to lymph nodes partially explains the early dissemination of bacteria from the entry site to various tissues and organs [53]. Dendritic cells in syphilis serve as a bridge between innate and adaptive immune responses, primarily through the presentation of treponemal antigens to T lymphocytes in lymph nodes and subsequent stimulation of T lymphocytes to differentiate and migrate to the bacterial entry site.

### 3.2. T Cells

In studies on *T. pallidum* infection conducted using a rabbit model, the appearance of lymphocytes reactive to *T. pallidum* correlates with the progression of mononuclear cell infiltration and macrophage activation at the sites of experimental inoculation [24]. Immunohistochemical (IHC) and RT-PCR analyses of biopsy samples taken from patients with primary and secondary syphilitic lesions demonstrate that syphilitic skin lesions also consist of lymphocytes and macrophages capable of expressing Th1 cytokine, IL-2, IFN-γ, and IL-12 mRNAs [24]. It has been shown that helper T lymphocytes outnumber cytolytic T lymphocytes in experimentally infected rabbit tissues [41] and in human primary syphilitic lesions [54]. However, in secondary syphilis, inflammatory infiltrates are characterized by a higher number of CD8+ T lymphocytes [54,55]. Observations made by Van Voorhis et al. [56], indicating that both perforin and granzyme B (markers of activation for these cells) are expressed in human syphilitic lesions during secondary syphilis, confirm that cytolytic T lymphocytes play a role in bacterial clearance during this stage of the disease [48,56]. Nevertheless, how CD8+ T lymphocytes are activated in the skin remains unclear. It should be noted that these lymphocytes typically respond to antigens presented via the major histocompatibility complex (MHC) class I pathway (the endogenous peptide presentation pathway) [57], which is generally not associated with the control of predominantly extracellular pathogens like *T. pallidum*. Thus, the role of these cells in responding to *T. pallidum* infection remains uncertain. However, it appears that mediators produced by CD8+ T lymphocytes present at the infection site significantly contribute to tissue destruction, which is reflected in the clinical presentation [58].

CD4+ T lymphocytes secrete cytokines, such as interferon gamma (IFN-γ) and interleukin 2 (IL-2), which stimulate macrophage activation, enhancing their ability to phagocytose and destroy bacteria [32]. These cytokines also exert a pro-inflammatory effect at the infection site, leading to the formation of the primary ulcer and partial containment of bacterial spread [9].

Meanwhile, regulatory T lymphocytes (Tregs) play a suppressive role during infection, dampening excessive inflammatory responses through the secretion of anti-inflammatory cytokines such as interleukin 10 (IL-10) and transforming growth factor-beta (TGF-β) [58]. This Treg activity reduces tissue damage on the one hand, but it may also facilitate bacterial survival in the later stages of infection, leading to chronic and latent infection [59].

There is also a subpopulation of CD4+ T cells known as Th17 cells, characterized by the production of interleukin 17 (IL-17A) [60]. Th17 cells are effector cells that respond to extracellular bacteria and are present in autoimmune diseases [61,62,63]. They are involved in the pathogenesis of numerous inflammatory and autoimmune disorders due to the broad distribution of IL-17 receptors [64]. Increasing evidence suggests that Th17 cells are crucial effectors in several chronic inflammatory diseases previously considered to be Th1-mediated, such as multiple sclerosis [65], rheumatoid arthritis [66], inflammatory bowel disease [67], systemic lupus erythematosus [68], and psoriasis [69]. These characteristics have led to syphilis being described as a disease mimicking autoimmune disorders, such as lupus, multiple sclerosis, psoriasis, or tabes dorsalis, among others [70].

It appears that Th17 cells do not play a significant role in the primary immune response. However, IL-17 levels are increased by CD8+ and NK cell responses to recruit neutrophils for bacterial phagocytosis. In secondary syphilis (SS), elevated concentrations of Th17 cells have been reported in venous blood. A study conducted by Stary et al. [55] showed that, among the dominant CD8+ T lymphocytes in the inflammatory infiltrate of typical secondary syphilitic skin lesions, most cells produce both IFN-γ and IL-17 [55]. In tertiary syphilis, the most severe tissue damage begins in the central nervous system (CNS) and cardiovascular system (CVS). Interestingly, at this stage of the disease, both CD8+ T cell and Th17 cell numbers are elevated.

Studies have shown that Th17 cells contribute to clearing the host of various pathogenic organisms (*Mycobacterium tuberculosis*, *Klebsiella pneumonia*, *Candida albicans,* and *Pneumocystis carinii*) [71,72,73,74,75]. On the other hand, Th17 also mediates strong immunopathology in chronic infection. The Th17 response to infection can involve both progression/chronic infection and protection [76,77,78,79]. Moreover, it has been postulated that Treg and Th17 cells are antagonistic to each other in order to maintain a balance in immune response mechanisms [63]. This balance may promote the differentiation of initial CD4+ T cells into Treg cells and limit the presence of Th17 cells, which leads to cellular immunosuppression and may ultimately cause chronic infection [63]. Another potent inhibitor of Th17 cells is IL-27, and studies have shown its significant inhibitory effect on T regulatory, Th1, Th2, and Th17 cells [80,81,82]. IL-27 prevents the evolution of pro-inflammatory Th17 cells by inhibiting expression of the Th17 transcription factor, thereby suppressing IL-17 production in naive T cells [76,80].

### 3.3. Macrophages

The role of macrophages in eliminating *T. pallidum* was first suggested when whole or partially degraded pathogens were identified in the phagocytic vacuoles of macrophages [83]. Subsequently, it was demonstrated that, in response to cytokines produced by T lymphocytes (mainly IFN-γ), macrophages migrate to the infection site, phagocytose, and kill *T. pallidum* [84]. Phagocytosis is more efficient if the pathogen is additionally coated with complement proteins, immunoglobulins, or antibodies directed against lipid antigens [85]. Interestingly, for reasons still unknown, some *T. pallidum* bacteria are resistant to opsonization and thus may not be phagocytosed by macrophages. It has been suggested that this subpopulation of bacteria may survive at the infection site and, if untreated, contribute to disease progression [33].

### 3.4. NK Cells

NK cells play a significant role in the early immune response to *T. pallidum* infection, although their function in syphilis pathogenesis is less understood compared to T lymphocytes. NK cells are activated in response to signals initiated by Pattern Recognition Receptors (PRRs) on dendritic cells and macrophages. As effector cells, NK cells are recruited to the site of infection by chemokines secreted by macrophages, such as CXCL9 and CXCL10, which are produced in response to infection. A key mechanism of NK cell action involves the secretion of interferon-gamma (IFN-γ), which stimulates the Th1 response and macrophage activation, enhancing NK cell capacity to phagocytose bacteria [32,59]. In addition to cytokine secretion, NK cells can eliminate infected host cells through degranulation-dependent mechanisms, thereby limiting bacterial dissemination [32,58].

NK cells also play a crucial role in modulating the intensity of the inflammatory response during *T. pallidum* infection. By secreting anti-inflammatory cytokines such as IL-10, they can reduce the tissue damage caused by excessive immune responses [51]. Moreover, through interactions with T regulatory cells (Tregs), NK cells influence the balance between pro-inflammatory and suppressive responses. Their activity is particularly important in the early stages of infection but may also contribute to autoimmune processes in advanced syphilis, where chronic stimulation of the immune system leads to host tissue destruction [9].

Although NK cells are not the primary actors in the immune mechanisms targeting *T. pallidum*, their ability to activate early, secrete IFN-γ, and influence other immune cells highlights their significant role in controlling the infection.

### 3.5. Cytokines

Cytokines are key mediators of the immune response in *T. pallidum* infection, playing a crucial role in activating immune cells, regulating inflammation, and coordinating the response to the infection. During *T. pallidum* infection, a variety of cytokines are secreted by immune cells, including macrophages, dendritic cells, T lymphocytes, and NK cells. Their production and function depend on the stage of infection.

In the early stages of *T. pallidum* infection, the immune response is dominated by pro-inflammatory cytokines secreted by phagocytic cells such as macrophages and dendritic cells in response to recognized pathogens. Interleukin 12 (IL-12) is produced, which stimulates the development of CD4+ T cells toward a Th1 response, thus initiating the process of bacterial elimination [9]. Another cytokine released is tumor necrosis factor-alpha (TNF-α), a potent pro-inflammatory factor that enhances the inflammatory response but can also contribute to tissue damage if produced in excess [86]. Interferon gamma (IFN-γ), mentioned repeatedly, is produced and secreted by T lymphocytes and NK cells. It stimulates macrophages to perform effective phagocytosis and destroy *T. pallidum* [58].

IL-17 produced by Th17 cells is a potent pro-inflammatory cytokine that plays a key role in the induction and development of tissue damage. IL-17 influences the increased production of ICAM-1, IL-6, and IL-8 and enhances the effects of cytokines such as IL-1β and TNF-α, which increases local inflammation and leads to inflammatory destruction [71,87,88]. Wang C. et al. [71] observed elevated levels of IL-17 in cerebrospinal fluid (CSF) in patients with neurosyphilis (NS). Interestingly, they also showed that IL-17 levels in CSF are positively correlated with VDRL titers and total protein in CSF in patients with NS. These findings suggest that IL-17 may be involved in central nervous system damage in patients with NS [71]. Also, a study by Pastuszczak et al. [89] showed that patients with NS had higher levels of IL-17 and IFN-γ in the CSF than those without NS [89]. IL-17 can disrupt connections in the vascular endothelium and activate its contractile mechanism, which can lead to blood-brain barrier (BBB) dysfunction [90]. These findings may suggest that IL-17 is involved in the tertiary stage of syphilis and that its range of action may cause more tissue damage in the CNS than its ability to clear *T. pallidum* [70]. The above evidence suggests that neurological damage in syphilis patients is associated with an increased CSF Th17/IL-17 response, and thus CSF IL-17 can be used to assess clinical outcomes of CNS syphilis treatment [71].

During the immune response, alongside the production of pro-inflammatory cytokines, anti-inflammatory cytokines are also produced. Among the most well-known are IL-10 and TGF-β, which play a vital role in regulating the intensity of the immune response to prevent excessive tissue damage.

In lesions typical of primary and secondary syphilis, elevated expression of IL-10 has been observed. IL-10 is one of the most important cytokines inhibiting immune and inflammatory responses [52]. This cytokine can be produced by various immune cells, primarily CD4+ CD25+ T regulatory cells (Tregs) and macrophages. IL-10 acts as an immunosuppressor, primarily by inhibiting activation of pro-inflammatory signals through suppression of the production and activity of inflammatory cytokines such as IL-1, IL-6, IL-12, and TNF-α. Additionally, reducing IFN-γ production prevents macrophages from effectively activating T lymphocytes [91,92]. Furthermore, IL-10 inhibits antigen presentation and the differentiation of T cells, B cells, NK cells, mast cells, and granulocytes [79,93]. On one hand, it is noted that weakened activation of cells producing anti-inflammatory cytokines in the early stages of infection can lead to an excessive, uncontrolled pro-inflammatory response. This can result in extensive tissue and organ damage. On the other hand, an excessive anti-inflammatory response can suppress the pro-inflammatory response, hindering pathogen elimination and predisposing to chronic or recurrent infections [94].

These immunosuppressive properties of IL-10 are exploited by some pathogens to survive in the host organism. Increased IL-10 production has been demonstrated in response to infections by *Plasmodium* spp. [95], *Leishmania* spp. [96], *Mycobacterium* [97], HIV [98], and HCV [99], correlating strongly with a tendency toward chronicity in these infections.

The second major anti-inflammatory cytokine is transforming growth factor-beta (TGF-β). TGF-β is essential in modulating the immune response, thereby promoting the development of Tregs, which suppress inflammation, ultimately reducing excessive tissue damage. However, on the other hand, it facilitates *T. pallidum* survival in the chronic phase of infection [32].

**Figure 2:**
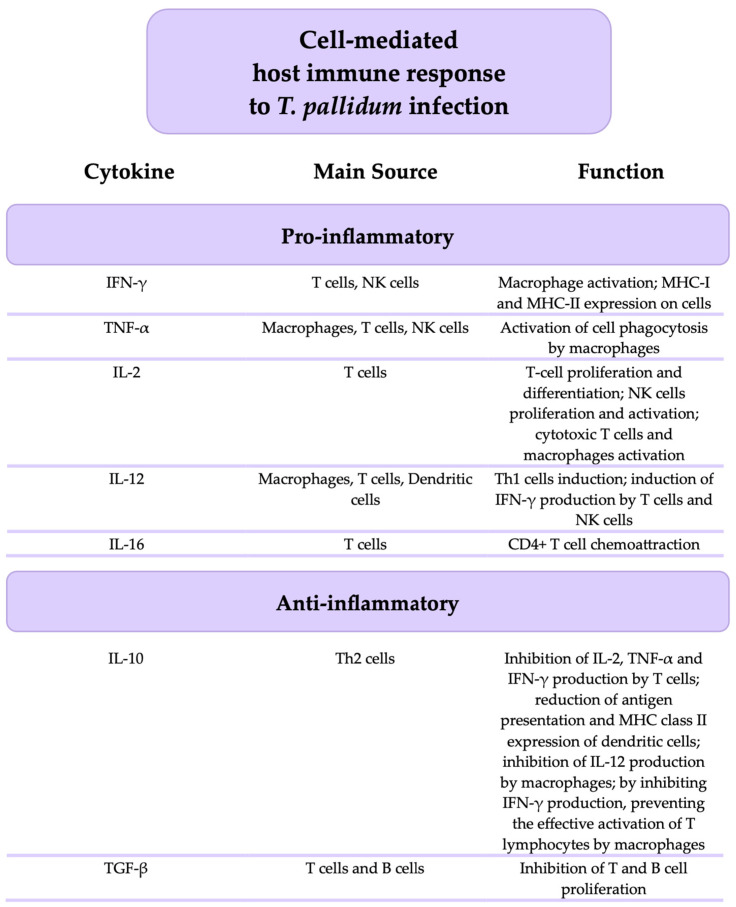
The principal pro-inflammatory and anti-inflammatory cytokines involved in the cell-mediated host immune response to *T. pallidum* infection.

Figure 2 presents the main pro-inflammatory and anti-inflammatory cytokines involved in the cell-mediated host immune response to *T. pallidum* infection, along with a brief description of the cells that produce them and their key functions.

### 3.6. The Role of the Vascular Endothelium in Cell-Mediated Immune Response to T. pallidum

The vascular endothelium is a critical natural barrier that separates the circulatory system from organs and tissues. It is also one of the first points of contact for pathogenic microorganisms. Consequently, the response of endothelial cells upon encountering a pathogen is thought to play a pivotal role in initiating the host immune response [100,101]. Endothelial cells actively participate in immune mechanisms by secreting cytokines to induce signaling pathways that increase or decrease inflammation. They also express adhesion receptors to recruit and enable leukocyte extravasation into organs and tissues during infection [100]. Moreover, endothelial cells secrete components of the extracellular matrix (ECM), which facilitate adhesion and communication between immune cells, pathogens, and the endothelium [100,102].

*T. pallidum* has the ability to penetrate easily through the vascular endothelium, as well as more restrictive barriers such as the blood-brain barrier (BBB) and the placental barrier. This capability leads to chronic and systemic infections that can persist for life if not treated with antibiotics [9,100,103].

Studies have shown that endothelial cells increase the secretion of pro-inflammatory cytokines, such as interleukin 6 (IL-6) and interleukin 8 (IL-8), as well as vascular endothelial growth factor (VEGF), after exposure to live *T. pallidum* [100]. Research by Waugh S. et al. [100] revealed that endothelial cells exposed to *T. pallidum* exhibit significantly altered expression of ECM components and cytoskeletal-regulating proteins, reduced expression of proteins involved in immune signaling, and differential expression of proteins associated with necroptotic cell death pathways [100]. The study also demonstrated that brain endothelial cells (BECs) exposed to *T. pallidum* both in vitro and in vivo increased the secretion of IL-6, IL-8, and VEGF while reducing the secretion of monocyte chemoattractant protein-1 (MCP-1) over 5 to 72 h [100]. These findings align with earlier studies showing increased IL-6 secretion from human dermal vascular smooth muscle cells (HDVSMCs) exposed to *T. pallidum* [104] and elevated serum IL-6 levels in individuals diagnosed with syphilis [105].

Elevated IL-6 and IL-8 secretion may facilitate pathogen dissemination by increasing vascular permeability, which is associated with reduced expression of tight junction proteins such as claudin, occludin, and VE-cadherin [100,106,107]. IL-6 also induces VEGF expression [108], and increased VEGF secretion has been observed in BECs exposed to *T. pallidum* in vitro and in vivo [100]. Notably, elevated VEGF levels have been documented in skin lesions of individuals with secondary syphilis [109].

Waugh S. et al. [100] reported reduced MCP-1 secretion in BECs exposed to in vivo *T. pallidum* and decreased CSF1 expression (Log2FC −0.45) in their proteomic analysis [100]. Additionally, they described novel antimicrobial peptides (AMPs) from *T. pallidum* that decrease MCP-1 secretion in THP-1 macrophage-differentiated cells under pro-inflammatory conditions [100,110]. MCP-1 is an important chemokine for monocyte recruitment, while CSF1 is a critical growth factor for monocyte/macrophage differentiation, survival, and proliferation [111,112].

Effective clearance of *T. pallidum* at infection sites involves the induction of a delayed-type hypersensitivity (DTH) response, mediated by CD4+ T cell infiltration and macrophage activation to phagocytose and kill *T. pallidum* [8,85]. Reduced MCP-1 and CSF1 secretion in BECs exposed to *T. pallidum* may impair monocyte migration to sites of *T. pallidum* colonization, enabling the pathogen to evade elimination and spread during the early stages of infection [100].

### 3.7. Keratinocytes Involved in Immune Response Against T. pallidum

Keratinocytes, being the main cells of the epidermis, form a barrier against physical, chemical, and microbiological agents. Importantly, they also produce a variety of cytokines, chemokines, and antimicrobial peptides that participate in the activation of immune system cells in response to infections and tissue repair [113,114,115]. They express multiple Toll-like receptors (TLRs), which allows them to recognize PAMPs and danger-associated molecular patterns (DAMPs). In the *T. pallidum* infection, Toll-like receptor 2 (TLR2), Toll-like receptor 4 (TLR4), and Toll-like receptor 5 (TLR5) [116,117,118,119,120,121] are crucial. TLRs also recruit the adaptor protein myeloid differentiation factor 88 (MyD88) to the receptors, which mediates the activation of mitogen-activated protein kinases (MAPKs) and nuclear factor-κB (NF-κB) signaling pathways, thereby promoting the production of pro-inflammatory cytokines [122]. MAPK signaling pathways can be stimulated by bacterial products and other factors to mediate signal transduction, induce production of pro-inflammatory cytokines, and promote the inflammatory response. In skin and mucosal cells, the NF-κB signaling pathway (κB is a key link between the innate and adaptive immune responses) regulates the expression of genes that promote the inflammatory response [122].

One of the PAMPs that interacts with TLR5 is *T. pallidum* flagellin. It is a subunit of flagellin and induces the expression of matrix metalloproteinases MMP-9 and MMP-13 in keratinocytes, which are involved in activation and regulation through the NF-κB and MAPK signaling pathways [118]. Flagellins also induce keratinocytes to produce IL-6 and IL-8 through the TLR2 pathway [122]. These findings suggest that the *T. pallidum* flagellins FlaB1, FlaB2, and FlaB3 may be crucial for inducing inflammation, which may contribute to skin inflammatory responses and keratinocyte-mediated ECM degradation during *T. pallidum* infection. Hence, the ability of flagellins to stimulate non-specific host resistance may be important for protection against *T. pallidum*. Unfortunately, MMP expression also promotes bacterial invasion by destroying tissue barriers [123].

## 4. HIV/Syphilis Co-Infection

Syphilis rates are high in PLWHIV. In the context of the significance of the cellular response in syphilis, this is interesting, as the immune response plays a major role in both diseases.

Knudsen et al. [124] studied samples from 36 patients with HIV/syphilis co-infection. The authors indicated an association between increased IL-10 levels and the primary stage of syphilis in HIV-infected patients, even though the CD4+ T cell response was low. IL-10 and TNF concentrations decreased after antibiotic treatment. In the following years, it was shown that syphilis co-infection in HIV-infected individuals can reverse the imbalance of Vδ1 and Vδ2 T cells in acute HIV infections. It has also been shown that chronically HIV-infected individuals have more IL-17 produced by T γδ cells than those with acute HIV infection, regardless of the stage of syphilis. Increased concentrations of IL-17 were associated with a higher percentage of neutrophils, suggesting that they may play a role in the progression of HIV and that they may represent a link between innate and acquired immunity [125]. Another interesting report was a 2017 study by Kenyon et al. [126]. The authors studied 79 people with HIV and 12 without HIV, both groups with newly diagnosed syphilis. They analyzed a set of cytokines before antibiotic treatment and 6 months after treatment. Differences were observed in the immune response to *T. pallidum* between HIV-infected and HIV-uninfected individuals, which was manifested by a tenfold higher level of IL-10 in the HIV-positive group. The authors did not observe an analogous increase in pro-inflammatory cytokines characteristic of the Th1 response. IL-10 concentrations decreased after treatment, but did not reach baseline. This may suggest that increased IL-10 concentrations act as a feedback response limiting inflammation. A complementary explanation may be that *T. pallidum* plays a role in increasing IL-10 levels, and this may have the effect of enhancing the immune suppressive response of Tregs. This can lead to a transition into chronic latent stages of infection.

## 5. Humoral Response to *T. pallidum* Infection

The process of elimination of bacteria from tissues seems to depend primarily on the cellular response, but the humoral response should also be mentioned, which is also part of the immune response to *T. pallidum* infection. Humoral antibodies are relatively ineffective in clearing syphilitic infections or in controlling the progression of lesions; secondary and tertiary disease follows (despite high antibody levels, tissues are continuously colonized by live spirochetes) if the cellular response is insufficiently effective [127].

The humoral response based on the production of antibodies by plasma cells in syphilis develops as in other infectious diseases. The antibodies produced are directed against lipid and protein antigens of *T. pallidum.* However, there is low recognition of the bacterium by antibodies, allowing the pathogen to multiply without activating the host immune system [127,128,129].

The most important mechanism in the humoral response appears to be the production of opsonins. Opsonins coat bacteria, which makes the pathogen able to be recognized by cells involved in the immune response (mainly macrophages) and eliminated by phagocytosis. IgM class antibodies are produced about two weeks after infection. The titers of these antibodies decrease during the natural course of the infection, but also after treatment. Four to five weeks after infection, IgG class antibodies are produced. In addition, anti-spirochete antibodies are involved in blocking the adhesion of *T. pallidum* to host cells and immobilizing them [130].

## 6. Limitations of Research on *T. pallidum*

Although *T. pallidum* was identified microscopically at the beginning of the 20th century, its inability to be cultured under laboratory conditions has greatly limited and delayed research on the immunopathological mechanisms of syphilis and the pathogenicity of *T. pallidum* [131]. As mentioned above, *T. pallidum* bacteria tolerate desiccation, elevated temperature, and ambient oxygen tension very poorly.

Another factor that makes research much more difficult is the lack of a well-developed mouse model. The main animals used in the preclinical model of syphilis are rabbits. This is due to the similarity of the pathological changes and immune response to infection observed in rabbits to those that occur in humans. However, the rabbit model is not ideal, as it presents difficulties in genetic manipulation [131]. In addition, appropriate immune reagents have not been established, making such models even more difficult to establish [9,131,132]. In recent years, however, the possibility of developing a mouse model has resurfaced. Previous studies have shown that *T. pallidum* can infect and persist in mice, but that infected mice do not develop skin lesions, as observed in other animal models [133,134]. A study by Lu S. et al. [134] in C57BL/6 mice provided information on the spread and pathogenesis of *T. pallidum* in a mouse model, as well as insights into the use of mice as a useful model for the study of syphilis, particularly asymptomatic syphilis without visible external lesions.

As can be seen, researchers working on *T. pallidum* face a large number of factors that make it difficult to conduct studies aimed at a comprehensive and accurate understanding of the immunopathological basis of syphilis.

## 7. Conclusions and Future Directions

The cell-mediated host immune response plays a key role in the infection caused by *T. pallidum*, the bacterium responsible for syphilis. In response to bacterial invasion, a series of mechanisms are activated, including the activation of immune cells such as macrophages, dendritic cells, T lymphocytes, NK cells, and cytotoxic T lymphocytes, all of which aim to clear tissues of the pathogen.

The main players in the response to *T. pallidum* infection are CD4+ T lymphocytes and the Th1 response, with a dominant role for IFN-γ. Early activation of T lymphocytes aids in bacterial elimination, while NK cells support the inflammatory response by eliminating infected cells. At the same time, anti-inflammatory cytokines such as IL-10 and TGF-β play an important role in maintaining optimal intensity of the immune response and preventing excessive tissue damage. The balance between pro-inflammatory and anti-inflammatory responses appears to be crucial for effective pathogen elimination and minimizing tissue damage in the host. In the chronic stages of syphilis, suboptimal regulation of the immune response may lead to long-term survival of *T. pallidum* in the host organism.

Despite the considerable difficulties in the study of *T. pallidum*, due mainly to its complex culture system requirements, the development of research and molecular biology techniques has made it possible in recent years to explore more deeply the morphology and pathophysiology of syphilis.

Although much is now known about the pathophysiology of syphilis, there is still much to be explained. Research into the role of the cellular immune response in *T. pallidum* infection provides valuable information that could assist in developing new therapeutic strategies, especially in cases of chronic infections and co-infections.

Despite the presence of a relatively cheap and accessible cure for syphilis in the form of antibiotics, it is undoubtedly the vision of the future to develop a vaccine against *T. pallidum*, which would be a good preventive mechanism against infection and, as a result, would help limit the spread of the disease.

As we presented in this review article, *T. pallidum* has a pronounced ability to evade the host immune response. This is due to the high antigenic variability of its surface proteins, as well as the activation of cells and cytokines with anti-inflammatory effects. This results in transition of the infection to a chronic state. It would undoubtedly be helpful to develop methods of immunomodulation, by which the mechanisms of the immune response could be strengthened, achieving effective clearance of *T. pallidum* from tissues.

## Data Availability

The raw data supporting the conclusions of this article will be made available by the authors on request.

## References

[1] Stamm L.V. (2016). Syphilis: Re-emergence of an old foe. Microb. Cell.

[2] Rowley J., Vander Hoorn S., Korenromp E., Low N., Unemo M., Abu-Raddad L.J., Chico R.M., Smolak A., Newman L., Gottlieb S. (2019). Chlamydia, gonorrhoea, trichomoniasis and syphilis: Global prevalence and incidence estimates 2016. Bull. World Health Organ..

[3] World Health Organization (2023). Sexually Transmitted Infections (STIs). https://www.who.int/news-room/fact-sheets/detail/sexually-transmitted-infections-(stis).

[4] World Health Organization (2024). Syphilis Cases Increase in the Americas. https://www.paho.org/en/news/22-5-2024-syphilis-cases-increase-americas.

[5] World Health Organization (2024). New Report Flags Major Increase in Sexually Transmitted Infections, Amidst Challenges in HIV and Hepatitis. https://www.who.int/news/item/21-05-2024-new-report-flags-major-increase-in-sexually-transmitted-infections---amidst-challenges-in-hiv-and-hepatitis.

[6] European Centre for Disease Prevention and Control Syphilis—Annual Epidemiological Report 2022. https://www.ecdc.europa.eu/en/publications-data/syphilis-annual-epidemiological-report-2022.

[7] Scurtu L.G., Jinga V., Simionescu O. (2022). Fascinating Molecular and Immune Escape Mechanisms in the Treatment of STIs (Syphilis, Gonorrhea, Chlamydia, and Herpes Simplex). Int. J. Mol. Sci..

[8] Carlson J.A., Dabiri G., Cribier B., Sell S. (2011). The immunopathobiology of syphilis: The manifestations and course of syphilis are determined by the level of delayed-type hypersensitivity. Am. J. Dermatopathol..

[9] LaFond R.E., Lukehart S.A. (2006). Biological basis for syphilis. Clin. Microbiol. Rev..

[10] Radolf J.D., Hazlett K.R.O., Lukehart S.A., Radolf J.D., Lukehart S.A. (2006). Pathogenesis of Syphilis. Pathogenic Treponemes: Cellular and Molecular Biology.

[11] Baughn R.E., Musher D.M. (2005). Secondary syphilitic lesions. Clin. Microbiol. Rev..

[12] Huang J., Lin S., Wan B., Zhu Y. (2018). A Systematic Literature Review of Syphilitic Hepatitis in Adults. J. Clin. Transl. Hepatol..

[13] Hook E.W. (2019). Syphilis. Lancet.

[14] Salazar J.C., Cruz A.R., Pope C.D., Valderrama L., Trujillo R., Saravia N.G., Radolf J.D. (2007). *Treponema pallidum* elicits innate and adaptive cellular immune responses in skin and blood during secondary syphilis: A flow-cytometric analysis. J. Infect. Dis..

[15] Cox D.L., Radolf J.D. (2016). *Treponema pallidum*: From pathogenesis to vaccines. Clin. Microbiol. Rev..

[16] Hook E.W., Marra C.M. (1992). Acquired syphilis in adults. N. Engl. J. Med..

[17] Wicher K., Wicher V., Abbruscato F., Baughn R.E. (2000). *Treponema pallidum* subsp. pertenue displays pathogenic properties different from those of *T. pallidum* subsp. pallidum. Infect. Immun..

[18] Centurion-Lara A., Arroll T., Castillo R., Shaffer J.M., Castro C., Van Voorhis W.C., Lukehart S.A. (1997). Conservation of the 15-kilodalton lipoprotein among *Treponema pallidum* subspecies and strains and other pathogenic treponemes: Genetic and antigenic analyses. Infect. Immun..

[19] Centurion-Lara A., Castro C., Castillo R., Shaffer J.M., Van Voorhis W.C., Lukehart S.A. (1998). The flanking region sequences of the 15-kDa lipoprotein gene differentiate pathogenic treponemes. J. Infect. Dis..

[20] Cameron C.E., Castro C., Lukehart S.A., Van Voorhis W.C. (1999). Sequence conservation of glycerophosphodiester phosphodiesterase among *Treponema pallidum* strains. Infect. Immun..

[21] Wicher K., Baughn R.E., Wicher V., Nakeeb S. (1992). Experimental congenital syphilis: Guinea pig model. Infect. Immun..

[22] Wicher K., Baughn R.E., Abbruscato F., Wicher V. (1999). Vertical transmission of *Treponema pallidum* to various litters and generations of guinea pigs. J. Infect. Dis..

[23] Baughn R.E., Wicher V., Jakubowski A., Wicher K. (1987). Humoral response in *Treponema pallidum*-infected guinea pigs. II. Circulating immune complexes and autoimmune responses. J. Immunol..

[24] Cruz A.R., Ramirez L.G., Zuluaga A.V., Pillay A., Abreu C., Valencia C.A., La Vake C., Cervantes J.L., Dunham-Ems S., Cartun R. (2012). Immune evasion and recognition of the syphilis spirochete in blood and skin of secondary syphilis patients: Two immunologically distinct compartments. PLoS Neglected Trop. Dis..

[25] Norgard M.V., Arndt L.L., Akins D.R., Curetty L.L., Harrich D.A., Radolf J.D. (1996). Activation of human monocytic cells by *Treponema pallidum* and Borrelia burgdorferi lipoproteins and synthetic lipopeptides proceeds via a pathway distinct from that of lipopolysaccharide but involves the transcriptional activator NF-kappa B. Infect. Immun..

[26] Sellati T.J., Bouis D.A., Kitchens R.L., Darveau R.P., Pugin J., Ulevitch R.J., Gangloff S.C., Goyert S.M., Norgard M.V., Radolf J.D. (1998). *Treponema pallidum* and Borrelia burgdorferi lipoproteins and synthetic lipopeptides activate monocytic cells via a CD14-dependent pathway distinct from that used by lipopolysaccharide. J. Immunol..

[27] Cameron C.E., Radolf J.D., Lukehart S.A. (2006). The *T. pallidum* outer membrane and outer membrane proteins. Pathogenic Treponema: Molecular and Cellular Biology.

[28] Bourell K.W., Schulz W., Norgard M.V., Radolf J.D. (1994). *Treponema pallidum* rare outer membrane proteins: Analysis of mobility by freeze-fracture electron microscopy. J. Bacteriol..

[29] Jones J.D., Bourell K.W., Norgard M.V., Radolf J.D. (1995). Membrane topology of Borrelia burgdorferi and *Treponema pallidum* lipoproteins. Infect. Immun..

[30] Desrosiers D.C., Anand A., Luthra A., Dunham-Ems S.M., LeDoyt M., Cummings M.A., Eshghi A., Cameron C.E., Cruz A.R., Salazar J.C. (2011). TP0326, a *Treponema pallidum* β-barrel assembly machinery A (BamA) orthologue and rare outer membrane protein. Mol. Microbiol..

[31] Cox D.L., Luthra A., Dunham-Ems S., Desrosiers D.C., Salazar J.C., Caimano M.J., Radolf J.D. (2010). Surface immunolabeling and consensus computational framework to identify candidate rare outer membrane proteins of *Treponema pallidum*. Infect. Immun..

[32] Radolf J.D., Tramont E.C., Salazar J.C. (2016). *Treponema pallidum*: New insights into pathogenesis and immunity. Nat. Rev. Microbiol..

[33] Lukehart S.A., Shaffer J.M., Baker-Zander S.A. (1992). A subpopulation of *Treponema pallidum* is resistant to phagocytosis: Possible mechanism of persistence. J. Infect. Dis..

[34] Moore M.W., Cruz A.R., LaVake C.J., Marzo A.L., Eggers C.H., Salazar J.C., Radolf J.D. (2007). Phagocytosis of Borrelia burgdorferi and *Treponema pallidum* potentiates innate immune activation and induces gamma interferon production. Infect. Immun..

[35] LaFond R.E., Molini B.J., Van Voorhis W.C., Lukehart S.A. (2006). Antigenic variation of TprK V regions abrogates specific antibody binding in syphilis. Infect. Immun..

[36] Sell S., Salman J., Norris S.J. (1985). Reinfection of chancre-immune rabbits with *Treponema pallidum*. I. Light and immunofluorescence studies. Am. J. Pathol..

[37] Ho E.L., Lukehart S.A. (2011). Syphilis: Using modern approaches to understand an old disease. J. Clin. Investig..

[38] Xia W., Zhao J., Su B., Jiao Y., Weng W., Zhang M., Wang X., Guo C., Wu H., Zhang T. (2021). Syphilitic infection impairs immunity by inducing both apoptosis and pyroptosis of CD4^+^ and CD8^+^ T lymphocytes. Innate Immun..

[39] Babolin C., Amedei A., Ozolins D., Zilevica A., D’Elios M.M., de Bernard M. (2011). TpF1 from *Treponema pallidum* activates inflammasome and promotes the development of regulatory T cells. J. Immunol..

[40] Pozzobon T., Facchinello N., Bossi F., Capitani N., Benagiano M., Di Benedetto G., Zennaro C., West N., Codolo G., Bernardini M. (2016). *Treponema pallidum* (syphilis) antigen TpF1 induces angiogenesis through the activation of the IL-8 pathway. Sci. Rep..

[41] Leader B.T., Godornes C., VanVoorhis W.C., Lukehart S.A. (2007). CD4+ lymphocytes and gamma interferon predominate in local immune responses in early experimental syphilis. Infect. Immun..

[42] Chung K.Y., Kim K.S., Lee M.G., Chang N.S., Lee J.B. (2002). *Treponema pallidum* induces up-regulation of interstitial collagenase in human dermal fibroblasts. Acta Derm. Venereol..

[43] Riley B.S., Oppenheimer-Marks N., Hansen E.J., Radolf J.D., Norgard M.V. (1992). Virulent *Treponema pallidum* activates human vascular endothelial cells. J. Infect. Dis..

[44] Tomson F.L., Conley P.G., Norgard M.V., Hagman K.E. (2007). Assessment of cell-surface exposure and vaccinogenic potentials of *Treponema pallidum* candidate outer membrane proteins. Microbes Infect..

[45] Edmondson D.G., Norris S.J. (2021). In Vitro Cultivation of the Syphilis Spirochete *Treponema pallidum*. Curr. Protoc..

[46] Lukehart S.A., Baker-Zander S.A., Sell S. (1980). Characterization of lymphocyte responsiveness in early experimental syphilis. I. In vitro response to mitogens and *Treponema pallidum* antigens. J. Immunol..

[47] Lukehart S.A., Baker-Zander S.A., Lloyd R.M., Sell S. (1980). Characterization of lymphocyte responsiveness in early experimental syphilis. II. Nature of cellular infiltration and *Treponema pallidum* distribution in testicular lesions. J. Immunol..

[48] Van Voorhis W.C., Barrett L.K., Koelle D.M., Nasio J.M., Plummer F.A., Lukehart S.A. (1996). Primary and secondary syphilis lesions contain mRNA for Th1 cytokines. J. Infect. Dis..

[49] Arroll T.W., Centurion-Lara A., Lukehart S.A., Van Voorhis W.C. (1999). T-Cell responses to *Treponema pallidum* subsp. pallidum antigens during the course of experimental syphilis infection. Infect. Immun..

[50] Cruz A.R., Pillay A., Zuluaga A.V., Ramirez L.G., Duque J.E., Aristizabal G.E., Fiel-Gan M.D., Jaramillo R., Trujillo R., Valencia C. (2010). Secondary syphilis in cali, Colombia: New concepts in disease pathogenesis. PLoS Neglected Trop. Dis..

[51] Radolf J.D., Deka R.K., Anand A., Šmajs D., Norgard M.V., Yang X.F. (2016). *Treponema pallidum*, the syphilis spirochete: Making a living as a stealth pathogen. Nat. Rev. Microbiol..

[52] Shin J.L., Chung K.Y., Kang J.M., Lee T.H., Lee M.G. (2004). The effects of *Treponema pallidum* on human dendritic cells. Yonsei Med. J..

[53] Bouis D.A., Popova T.G., Takashima A., Norgard M.V. (2001). Dendritic cells phagocytose and are activated by *Treponema pallidum*. Infect. Immun..

[54] Engelkens H.J., ten Kate F.J., Judanarso J., Vuzevski V.D., van Lier J.B.H., Godschalk J.C., van der Sluis J.J., Stolz E. (1993). The localisation of treponemes and characterisation of the inflammatory infiltrate in skin biopsies from patients with primary or secondary syphilis, or early infectious yaws. Sex. Transm. Infect..

[55] Stary G., Klein I., Bruggen M.C., Kohlhofer S., Brunner P.M., Spazierer D., Müllauer L., Petzelbauer P., Stingl G. (2010). Host defense mechanisms in secondary syphilitic lesions: A role for IFN-gamma-/IL-17-producing CD8+ T cells?. Am. J. Pathol..

[56] Van Voorhis W.C., Barrett L.K., Nasio J.M., Plummer F.A., Lukehart S.A. (1996). Lesions of primary and secondary syphilis contain activated cytolytic T cells. Infect. Immun..

[57] Rock K.L. (1996). A new foreign policy: MHC class I molecules monitor the outside world. Immunol. Today.

[58] Yang C.H., Lin Y.H. (2020). Immunological aspects of syphilis. J. Formos. Med. Assoc..

[59] Ghanem K.G. (2018). The changing epidemiology of syphilis. JAMA.

[60] Miossec P., Korn T., Kuchroo V.K. (2009). Interleukin-17 and type 17 helper T cells. N. Engl. J. Med..

[61] Bedoya S.K., Lam B., Lau K., Larkin J. (2013). Th17 Cells in Immunity and Autoimmunity. Clin. Dev. Immunol..

[62] Yasuda K., Takeuchi Y., Hirota K. (2019). The pathogenicity of Th17 cells in autoimmune diseases. Semin. Immunopathol..

[63] Zhao J., Ma J., Zhang X., Li Q., Yang X. (2016). Equilibrium of Treg/Th17 cells of peripheral blood in syphilitic patients with sero-resistance. Exp. Ther. Med..

[64] Ishigame H., Kakuta S., Nagai T., Kadoki M., Nambu A., Komiyama Y., Fujikado N., Tanahashi Y., Akitsu A., Kotaki H. (2009). Differential roles of interleukin-17A and -17F in host defense against mucoepithelial bacterial infection and allergic responses. Immunity.

[65] Kebir H., Kreymborg K., Ifergan I., Dodelet-Devillers A., Cayrol R., Bernard M., Giuliani F., Arbour N., Becher B., Prat A. (2007). Human TH17 lymphocytes promote blood-brain barrier disruption and central nervous system inflammation. Nat. Med..

[66] Shen H., Goodall J.C., Hill Gaston J.S. (2009). Frequency and phenotype of peripheral blood Th17 cells in ankylosing spondylitis and rheumatoid arthritis. Arthritis Rheum..

[67] Fujino S., Andoh A., Bamba S., Ogawa A., Hata K., Araki Y., Bamba T., Fujiyama Y. (2003). Increased expression of interleukin 17 in inflammatory bowel disease. Gut.

[68] Wong C.K., Lit L.C., Tam L.S., Li E.K., Wong P.T., Lam C.W. (2008). Hyperproduction of IL-23 and IL-17 in patients with systemic lupus erythematosus: Implications for Th17-mediated inflammation in auto-immunity. Clin. Immunol..

[69] Kagami S., Rizzo H.L., Lee J.J., Koguchi Y., Blauvelt A. (2010). Circulating Th17, Th22, and Th1 cells are increased in psoriasis. J. Investig. Dermatol..

[70] Hernández-Pliego A., Vergara-Ortega D.N., Herrera-Ortíz A., Toledano-Jaimes C., Esquivel-Guadarrama F.R., Sánchez-Alemán M.Á. (2022). IL-10 and IL-17 as Progression Markers of Syphilis in People Living with HIV: A Systematic Review. Biomolecules.

[71] Wang C., Zhu L., Gao Z., Guan Z., Lu H., Shi M., Gao Y., Xu H., Yang X.F., Zhou P. (2014). Increased interleukin-17 in peripheral blood and cerebrospinal fluid of neurosyphilis patients. PLoS Neglected Trop. Dis..

[72] Chatterjee S., Dwivedi V.P., Singh Y., Siddiqui I., Sharma P., Van Kaer L., Chattopadhyay D., Das G. (2011). Early secreted antigen ESAT-6 of Mycobacterium tuberculosis promotes protective T helper 17 cell responses in a toll-like receptor-2-dependent manner. PLoS Pathog..

[73] Curtis M.M., Way S.S. (2009). Interleukin-17 in host defence against bacterial, mycobacterial and fungal pathogens. Immunology.

[74] Rudner X.L., Happel K.I., Young E.A., Shellito J.E. (2007). Interleukin-23 (IL-23)-IL-17 cytokine axis in murine Pneumocystis carinii infection. Infect. Immun..

[75] Saijo S., Ikeda S., Yamabe K., Kakuta S., Ishigame H., Akitsu A., Fujikado N., Kusaka T., Kubo S., Chung S.-H. (2010). Dectin-2 recognition of alpha-mannans and induction of Th17 cell differentiation is essential for host defense against Candida albicans. Immunity.

[76] Zhao W., Luo H. (2023). Investigation of the role of interleukin 27 in the immune regulation of Treg and Th17 cells in neurosyphilis patients. Folia Neuropathol..

[77] Afzali B., Lombardi G., Lechler R.I., Lord G.M. (2007). The role of T helper 17 (Th17) and regulatory T cells (Treg) in human organ transplantation and autoimmune disease. Clin. Exp. Immunol..

[78] Kryczek I., Wei S., Zou L., Altuwaijri S., Szeliga W., Kolls J., Chang A., Zou W. (2007). Cutting edge: Th17 and regulatory T cell dynamics and the regulation by IL-2 in the tumor microenvironment. J. Immunol..

[79] Pastuszczak M., Jakiela B., Jaworek A.K., Wypasek E., Zeman J., Wojas-Pelc A. (2015). Association of Interleukin-10 promoter polymorphisms with neurosyphilis. Hum. Immunol..

[80] Neufert C., Becker C., Wirtz S., Fantini M.C., Weigmann B., Galle P.R., Neurath M.F. (2007). IL-27 controls the development of inducible regulatory T cells and Th17 cells via differential effects on STAT1. Eur. J. Immunol..

[81] Kalliolias G.D., Ivashkiv L.B. (2008). IL-27 activates human monocytes via STAT1 and suppresses IL-10 production but the inflammatory functions of IL-27 are abrogated by TLRs and p38. J. Immunol..

[82] Stumhofer J.S., Hunter C.A. (2008). Advances in understanding the anti-inflammatory properties of IL-27. Immunol. Lett..

[83] Sell S., Baker-Zander S., Powell H.C. (1982). Experimental syphilitic orchitis in rabbits: Ultrastructural appearance of *Treponema pallidum* during phagocytosis and dissolution by macrophages in vivo. Lab. Investig..

[84] Lukehart S.A., Miller J.N. (1978). Demonstration of the in vivo phagocytosis of *Treponema pallidum* by Rabbit peritoneal macrophages. J. Immunol..

[85] Baker-Zander S.A., Lukehart S.A. (1992). Macrophage-mediated killing of opsonized *Treponema pallidum*. J. Infect. Dis..

[86] Cameron C.E., Lukehart S.A. (2004). *Treponema pallidum*: Surface and subsurface proteins in the pathogenesis of syphilis. Microbes Infect..

[87] Katz Y., Nadiv O., Beer Y. (2001). Interleukin-17 enhances tumor necrosis factor alpha-induced synthesis of interleukins 1,6, and 8 in skin and synovial fibroblasts: A possible role as a “fine-tuning cytokine” in inflammation processes. Arthritis Rheum..

[88] LeGrand A., Fermor B., Fink C., Pisetsky D.S., Weinberg J.B., Vail T.P., Guilak F. (2001). Interleukin-1, tumor necrosis factor alpha, and interleukin-17 synergistically up-regulate nitric oxide and prostaglandin E2 production in explants of human osteoarthritic knee menisci. Arthritis Rheum..

[89] Pastuszczak M., Jakiela B., Wielowieyska-Szybinska D., Jaworek A.K., Zeman J., Wojas-Pelc A. (2013). Elevated cerebrospinal fluid interleukin-17A and interferon-γ levels in early asymptomatic neurosyphilis. Sex. Transm. Dis..

[90] Huppert J., Closhen D., Croxford A., White R., Kulig P., Pietrowski E., Bechmann I., Becher B., Luhmann H.J., Waisman A. (2010). Cellular mechanisms of IL-17-induced blood-brain barrier disruption. FASEB J..

[91] Tabor D.R., Kiel D.P., Jacobs R.F. (1987). Cyclophosphamide-sensitive activity of suppressor T-cells during treponemal infection. Immunology.

[92] Borish L. (1998). IL-10: Evolving concepts. J. Allergy Clin. Immunol..

[93] Groux H., Bigler M., de Vries J.E., Roncarolo M.G. (1998). Inhibitory and stimulatory effects of IL-10 on human CD8+ T cells. J. Immunol..

[94] Chatila T. (2005). Role of regulatory T cells in human diseases. J. Allergy Clin. Immunol..

[95] Medina T.S., Costa S.P., Oliveira M.D., Ventura A.M., Souza J.M., Gomes T.F., Vallinoto A.C., Póvoa M.M., Silva J.S., Cunha M.G. (2011). Increased interleukin-10 and interferon-γ levels in Plasmodium vivax malaria suggest a reciprocal regulation which is not altered by IL-10 gene promoter polymorphism. Malar. J..

[96] Reed S.G., Brownell C.E., Russo D.M., Silva J.S., Grabstein K.H., Morrissey P.J. (1994). IL-10 mediates susceptibility to Trypanosoma cruzi infection. J. Immunol..

[97] Roque S., Nobrega C., Appelberg R., Correia-Neves M. (2007). IL-10 underlies distinct susceptibility of BALB/c and C57BL/6 mice to Mycobacterium avium infection and influences efficacy of antibiotic therapy. J. Immunol..

[98] Clerici M., Wynn T.A., Berzofsky J.A., Blatt S.P., Hendrix C.W., Sher A., Coffman R.L., Shearer G.M. (1994). Role of interleukin-10 in T helper cell dysfunction in asymptomatic individuals infected with the human immunodeficiency virus. J. Clin. Investig..

[99] Accapezzato D., Francavilla V., Paroli M., Casciaro M., Chircu L.V., Cividini A., Abrignani S., Mondelli M.U., Barnaba V. (2004). Hepatic expansion of a virus-specific regulatory CD8(+) T cell population in chronic hepatitis C virus infection. J. Clin. Investig..

[100] Waugh S., Ranasinghe A., Gomez A., Houston S., Lithgow K.V., Eshghi A., Fleetwood J., Conway K.M.E., Reynolds L.A., Cameron C.E. (2023). Syphilis and the host: Multi-omic analysis of host cellular responses to *Treponema pallidum* provides novel insight into syphilis pathogenesis. Front. Microbiol..

[101] Lemichez E., Lecuit M., Nassif X., Bourdoulous S. (2010). Breaking the wall: Targeting of the endothelium by pathogenic bacteria. Nat. Rev. Microbiol..

[102] Tomlin H., Piccinini A.M. (2018). A complex interplay between the extracellular matrix and the innate immune response to microbial pathogens. Immunology.

[103] Salazar J.C., Rathi A., Michael N.L., Radolf J.D., Jagodzinski L.L. (2007). Assessment of the kinetics of *Treponema pallidum* dissemination into blood and tissues in experimental syphilis by real-time quantitative PCR. Infect. Immun..

[104] Gao Z.X., Liu L.L., Lin L.R., Tong M.L., Liu F., Yang T.C. (2019). *Treponema pallidum* induces the secretion of HDVSMC inflammatory cytokines to promote the migration and adhesion of THP-1 cells. Front. Cell. Infect. Microbiol..

[105] Yan Y., Wang J., Qu B., Zhang Y., Wei Y., Liu H., Wu C. (2017). CXCL13 and TH1/Th2 cytokines in the serum and cerebrospinal fluid of neurosyphilis patients. Medicine.

[106] Paul R., Koedel U., Winkler F., Kieseier B.C., Fontana A., Kopf M., Hartung H.P., Pfister H.W. (2003). Lack of IL-6 augments inflammatory response but decreases vascular permeability in bacterial meningitis. Brain.

[107] Blecharz-Lang K.G., Wagner J., Fries A., Nieminen-Kelhä M., Rösner J., Schneider U.C., Vajkoczy P. (2018). Interleukin 6-Mediated Endothelial Barrier Disturbances Can Be Attenuated by Blockade of the IL6 Receptor Expressed in Brain Microvascular Endothelial Cells. Transl. Stroke Res..

[108] Adachi Y., Aoki C., Yoshio-Hoshino N., Takayama K., Curiel D.T., Nishimoto N. (2006). Interleukin-6 induces both cell growth and VEGF production in malignant mesotheliomas. Int. J. Cancer.

[109] Macaron N.C., Cohen C., Chen S.C., Arbiser J.L. (2003). Cutaneous lesions of secondary syphilis are highly angiogenic. J. Am. Acad. Dermatol..

[110] Houston S., Schovanek E., Conway K.M.E., Mustafa S., Gomez A., Ramaswamy R., Haimour A., Boulanger M.J., Reynolds L.A., Cameron C.E. (2022). Identification and Functional Characterization of Peptides with Antimicrobial Activity from the Syphilis Spirochete, *Treponema pallidum*. Front. Microbiol..

[111] Deshmane S.L., Kremlev S., Amini S., Sawaya B.E. (2009). Monocyte chemoattractant protein-1 (MCP-1): An overview. J. Interf. Cytokine Res..

[112] Sehgal A., Irvine K.M., Hume D.A. (2021). Functions of macrophage colony-stimulating factor (CSF1) in development, homeostasis, and tissue repair. Semin. Immunol..

[113] Nestle F.O., Di Meglio P., Qin J.Z., Nickoloff B.J. (2009). Skin immune sentinels in health and disease. Nat. Rev. Immunol..

[114] Chieosilapatham P., Kiatsurayanon C., Umehara Y., Trujillo-Paez J.V., Peng G., Yue H., Nguyen L.T.H., Niyonsaba F. (2021). Keratinocytes: Innate immune cells in atopic dermatitis. Clin. Exp. Immunol..

[115] Jiang Y., Tsoi L.C., Billi A.C., Ward N.L., Harms P.W., Zeng C., Maverakis E., Kahlenberg J.M., Gudjonsson J.E. (2020). Cytokinocytes: The diverse contribution of keratinocytes to immune responses in skin. JCI Insight.

[116] Luo X., Gao Z.X., Lin S.W., Tong M.L., Liu L.L., Lin L.R., Ke W.J., Yang T.C. (2020). Recombinant *Treponema pallidum* protein Tp0136 promotes fibroblast migration by modulating MCP-1/CCR2 through TLR4. J. Eur. Acad. Dermatol. Venereol..

[117] Xu M., Xie Y., Jiang C., Xiao Y., Kuang X., Wen Y., Tan Y., Tan M., Zhao F., Zeng T. (2017). *Treponema pallidum* flagellins elicit proinflammatory cytokines from human monocytes via TLR5 signaling pathway. Immunobiology.

[118] Jiang C., Xu M., Kuang X., Xiao J., Tan M., Xie Y., Xiao Y., Zhao F., Wu Y. (2017). *Treponema pallidum* flagellins stimulate MMP-9 and MMP-13 expression via TLR5 and MAPK/NF-κB signaling pathways in human epidermal keratinocytes. Exp. Cell Res..

[119] Huang T., Yang J., Zhang J., Ke W., Zou F., Wan C., Wang L., Zhang X., Liang F., Mei S. (2020). MicroRNA-101-3p Downregulates TLR2 Expression, Leading to Reduction in Cytokine Production by *Treponema pallidum*-Stimulated Macrophages. J Investig. Dermatol..

[120] Xie Y., Xu M., Xiao Y., Liu Z., Jiang C., Kuang X., Wang C., Wu H., Peng J., Li C. (2017). *Treponema pallidum* flagellin FlaA2 induces IL-6 secretion in THP-1 cells via the Toll-like receptor 2 signaling pathway. Mol. Immunol..

[121] Peng R.R., Shang S.X., Zhao L.S., Long F.Q. (2019). MiR-216a-5p-containing exosomes suppress rTp17-induced inflammatory response by targeting TLR4. Biosci. Rep..

[122] Luo Y., Xie Y., Chen J., Zhou J., Zhao F., Liu S., Zeng T., Xu M., Xiao Y. (2022). *Treponema pallidum* FlaA2 inducing the release of pro-inflammatory cytokines is mediated via TLR2 in keratinocytes. Microb. Pathog..

[123] Lee Y., Kim H., Kim S., Kim K.H., Chung J.H. (2010). Activation of toll-like receptors 2, 3 or 5 induces matrix metalloproteinase-1 and -9 expression with the involvement of MAPKs and NF-kappaB in human epidermal keratinocytes. Exp. Dermatol..

[124] Knudsen A., Benfield T., Kofoed K. (2009). Cytokine expression during syphilis infection in HIV-1-infected individuals. Sex. Transm. Dis..

[125] Li Z., Lu X., Hu Z., Luo Z., Jiang W., Wu H., Gao Y., Yan J., Zhang Q., Song A. (2017). Syphilis Infection Differentially Regulates the Phenotype and Function of γδ T Cells in HIV-1-Infected Patients Depends on the HIV-1 Disease Stage. Front. Immunol..

[126] Kenyon C., Osbak K.K., Crucitti T., Kestens L. (2017). The immunological response to syphilis differs by HIV status; a prospective observational cohort study. BMC Infect. Dis..

[127] Campo J.J., Romeis E., Oberai A., Pablo J.V., Hung C., Teng A.A., Shandling A.D., Phan A., Haynes A.M., Giacani L. (2024). A novel pan-proteome array for high-throughput profiling of the humoral response to *Treponema pallidum*. iScience.

[128] Blanco D.R., Champion C.I., Dooley A., Cox D.L., Whitelegge J.P., Faull K., Lovett M.A. (2005). A monoclonal antibody that conveys in vitro killing and partial protection in experimental syphilis binds a phosphorylcholine surface epitope of *Treponema pallidum*. Infect. Immun..

[129] Blanco D.R., Miller J.N., Lovett M.A. (1997). Surface antigens of the syphilis spirochete and their potential as virulence determinants. Emerg. Infect. Dis..

[130] Blanco D.R., Miller J.N., Hanff P.A. (1984). Humoral immunity in experimental syphilis: The demonstration of IgG as a treponemicidal factor in immune rabbit serum. J. Immunol..

[131] Salazar J.C., Hazlett K.R., Radolf J.D. (2002). The immune response to infection with *Treponema pallidum*, the stealth pathogen. Microbes Infect..

[132] Belkum A.V. (2007). Pathogenic Treponema: Molecular and cellular biology. J. Microbiol. Methods.

[133] Folds J.D., Rauchbach A.S., Shores E., Saunders J.M. (1983). Evaluation of the inbred mouse as a model for experimental *Treponema pallidum* infection. Scand. J. Immunol..

[134] Lu S., Zheng K., Wang J., Xu M., Xie Y., Yuan S., Wang C., Wu Y. (2021). Characterization of *Treponema pallidum* Dissemination in C57BL/6 Mice. Front. Immunol..

